# Supplementary figures and images for: Robotically Assisted Surgery in Children—A Perspective
Source: Children (Basel). 2022 Jun 6;9(6):839. doi: 10.3390/children9060839 (PMC9221697; doi:10.3390/children9060839)

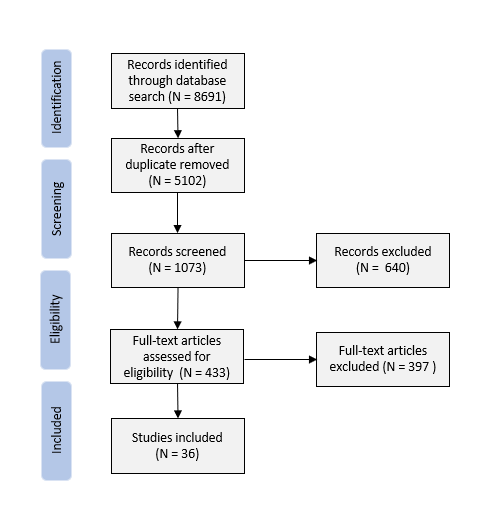

Supplement: Supplementary file 1 [file children-09-00839-s001.zip › children-1626121-supplementary.png]
